# Gender differences in the use of health care in China: cross-sectional analysis

**DOI:** 10.1186/1475-9276-13-8

**Published:** 2014-01-30

**Authors:** Yan Song, Ying Bian

**Affiliations:** 1 Institute of Chinese Medical Sciences, University of Macau, Av. Padre Tomás Pereira Taipa, Macau, China

**Keywords:** Gender differences, Inequity, Hospitalization, Use of health care, China

## Abstract

**Introduction:**

Differences between women and men in education, employment, political and economic empowerment have been well-documented in China due to the long traditional culture that male is superior to female. This study is to explore whether the similar gender differences exist in the use of health care by analyzing hospital admission, duration of hospitalization and medical expense of both genders in a Chinese hospital.

**Methods:**

This cross-sectional study evaluated the gender differences in clinical and epidemiologic characteristics of patients who were admitted for any reason to hospital in Zhuhai Special Economic Zone, Southern China, from January 1, 2003, through December 31, 2009. Chi-square test was used to calculate differences between proportions and the t test was used to test differences between means.

**Results:**

A total of 156,887 patients were recruited in the analysis, with a male/female ratio of 1.1:1.0. The average age and the duration of hospitalization were significantly greater among men (p < 0.05). A larger proportion of hospitalized female underwent surgery compared to male (p < 0.05). The total medical expense per inpatient indicated important differences between genders, with higher expenditures observed among men (p < 0.05). Furthermore, gender differences were observed in length of hospitalization and medical expense for five common conditions respectively and most differences favoring men were significant (p < 0.05) while differences favoring women were not significant (p > 0.05). Among all the self-paid patients, men were also superior in all investigating variables compared with women.

**Conclusions:**

Gender differences in the use of health care do occur in China. Despite of demographic factors, the differences between female and male can be in part explained by social power relations. China should increase attention to gender and equity in health.

## Introduction

Good health and equal access to high quality care—regardless of age, gender, geographic area, social status, or ethnic background–are important goals for health services in all countries. While socio-economically derived differences in health and use of health care was once a popular subject of study, gender-based differences, considered as an explanatory variable, recently has been paid more attention to [1,2]. Many existing researches suggested women, compared to men, make greater use of health-care services [3-6]. Among them, various explanations have been postulated and tested the women’s greater need resulting from their worse state of health (greater morbidity, worse perception of health, worse health-related quality of life, and greater degree of disability compared to men), the different social construction of the diseases (roles, attitudes, beliefs and behaviors of men and women when they are sick or concerned with ill-health), which leads to different processes for seeking health care and differences in the use of health care between women and men [4,5].

According to the latest National Survey on Health Service in China, both two-week morbidity rate and morbidity rate of chronic diseases of female were higher than those of male [7]. Given that the worse health status of female in China, the greater use of health care that female should make compared to men. However, with the long traditional culture that male are superior to female in China, men are in the dominant position in almost every field of social life. Differences between female and male in education, employment, political and economic empowerment have been well-documented [8,9]. Although the social position of female has been improved from the establishment of P. R. China in 1949, it is still impossible to iron out the old gender norms in such a short time. So in light of such gender gaps, some have expressed concerns that women might have less access to and use health care and medicines than men. However, there has been little empirical data showing gender-specific differences in health care in China. The report of National Health Services Survey only revealed the proportion of female having physician contact within two weeks was higher than that of male by about 20%. And female had higher hospitalization rate (7.6%) compared to male (6%) [7]. There is still inadequate information to reflect the characteristics of healthcare use by gender.

Therefore, the aim of the present study was to analyze hospital admission, duration of hospitalization and medical expense of both genders in a Chinese hospital and to compare the differences between them. The findings could provide more evidence to suggest whether the similar gender differences exist in the use of health care in China.

## Methods

### Study setting

This cross-sectional study evaluated gender differences in clinical and epidemiologic characteristics of patients who were admitted for any reason to a general hospital in Zhuhai Special Economic Zone, China. It is a teaching hospital affiliated to the China Ministry of Health and an essential part of the outstanding medical care system in Southern China [10]. Each year more than 20,000 patients go for inpatient treatments and a total of 450,000 people visit outpatient departments.

### Data collection

Data of male and female patients from January 1, 2003, through December 31, 2009 were collected from hospital medical record management system and the collection was anonymous (name or identifying information of the patients, general practitioners, and pharmacists were not obtained). The treatments included medical and surgical therapies. The research protocol was approved by the Research Ethics Committee of Institute of Chinese Medical Sciences, University of Macau.

### Study variable

Hospital admission, duration of hospitalization and medical expense (daily and during the total stay) were employed to analyze the use of health care. The available control variables in this study included age, employment status, health insurance coverage and cause of disease. The variables are possible contributing factors to gender differences in the use of health care. Previous researches revealed the health care utilization pattern was age-specific, usually high during infancy, low during childhood and rising thereafter, especially during old age [11]. And the health insurance coverage could increase the use of health services [12,13]. In addition, the self-paid patients are always with high levels of out-of-pocket payments. So the use of health care among the self-paid patient group may reveal larger gender differences, if it is affected by the traditional culture that male are superior to female. Finally, differences in duration and cost were analyzed within each of the five main causes of disease (ICD-10 code).

### Statistical analysis

Epidemiological and clinical data were entered into a Microsoft Office Excel 2007 database. Statistical analysis was performed with IBM SPSS version 19.0. Continuous variables were given as mean, median and range. Chi-square test was used to calculate differences between proportions and the t test was used to test differences between means. Additionally, during analysis, the total medical expense per inpatient was transformed into natural logarithms of the observation value to address the positive skew of the expenditure data. Results with a 2-sided *p* value <0.05 were considered statistically significant.

## Results

A total of 156,887 patients who underwent hospitalizations were employed for analysis in the seven-year period. The main characteristics of these individuals by gender are shown in Table 1. Comparison of the variables between female and male during hospitalization revealed differences with clinical and statistical significance.

**Table 1:** Main characteristics of all patients admitted to hospital by gender, 2003-2009

**Variable**	**Male (n = 82,175)**	**Female (n = 74,712)**	***p *** **value**
Age, year			
Mean (SD)	46.91 (21.16)	44.25 (19.23)	<0.01^*^
Median (range)	48 (0–142)	44 (0–104)	
Age range, no. (%)			
0-	1664 (2.02)	1073 (1.44)	
1-	2446 (2.98)	1520 (2.03)	
5-	2753 (3.35)	1590 (2.13)	
15-	5614 (6.83)	6471 (8.66)	
25-	10082 (12.27)	14239 (19.06)	
35-	13832 (16.83)	13386 (17.92)	
45-	12995 (15.81)	12566 (16.82)	
55-	12774 (15.54)	11244 (15.05)	
65-	20015 (24.36)	12623 (16.90)	
Duration of hospitalization, day			
Mean (SD)	11.84 (16.33)	10.31 (11.84)	<0.01^*^
Median (Vmin-Vmax)	9.00 (1–961)	8.00 (1–488)	
Medical expenses, yuan			
Percent %	57.63	42.37	
Mean (SD)	8806.31 (20539.23)	7122.56 (14998.65)	<0.01^*^
Surgical therapies (%)	37.16	43.96	<0.01^†^
Self-paid (%)	42.40	47.05	<0.01^†^
Occupation status, no. (%)			<0.01^†^
Government employee	30452 (37.06)	21653 (28.98)	
Private employee	13173 (16.03)	14382 (19.25)	
Self-employed	3121 (3.80)	1381 (1.85)	
Unemployed	28566 (34.76)	33113 (44.32)	
Children	6863 (8.35)	4183 (5.60)	

Vmin = minimum value; Vmax = maximum value.

^*^t test.

^†^Chi square test.

There was a slightly larger proportion of male patients (82,175; 52.40%) compared to female ones (74,712; 47.60%), resulting in a 1.1:1.0 ratio. The median age of study population was 46 years. 7.04% were children (under 15 years old). Male were significantly older at admission (p < 0.01). Fewer female were admitted to hospital than male across all age groups, especially in the group aged <15 and group aged >65, where the male/female ratios were 1.6:1.0 for both. However, there was one exception that in the patient group aged from 15 to 35 years, more female were admitted to hospital. Furthermore, a larger proportion of hospitalized female underwent surgery compared to male (42.87% vs. 37.16%, respectively; p = 0.00). Significant difference was observed in the employment status between genders (p < 0.01). Most male patients (37.06%) were government employees, while the unemployed constituted the largest proportion of female patients.

**Figure 1:**
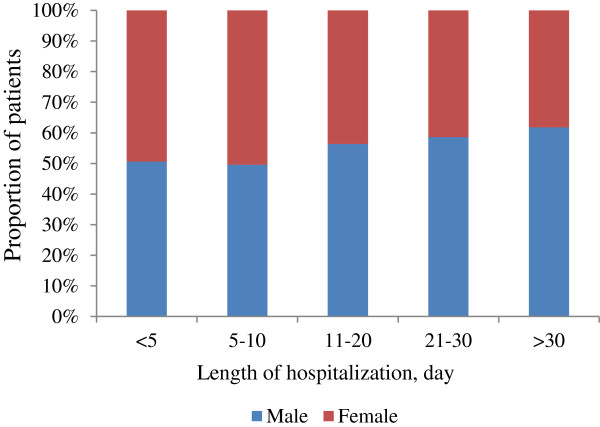
Hospital admissions by gender, by length of hospitalization.

Overall, men had a significantly longer duration of hospitalization than women (mean [SD] duration, 11.84[16.33] vs. 10.31[11.84] days, respectively; p = 0.00). And in prolonged hospitalization, a more obvious difference was observed between female and male (Figure 1). The number of male who stayed in hospital for ten or more days was 34.57% higher than that of female. And the number of male who stayed for 31 or more days was 61.93% higher than female. Also, the total medical expense per inpatient indicated significant differences between genders, with higher expenditures observed among men compared to women (t = 18.66, p = 0.00). For children, significant differences on the indicators favoring boys were only observed in the group aged <1 year. However, in any given group aged 15 years and above, male had a longer duration of hospitalization and spent more on hospitalization compared to female (p < 0.05 for all).

The top five causes of disease in hospital were in turn essential hypertension (I10 05, 3.06%), maintenance chemotherapy (Z51.201, 2.73%), atherosclerotic heart disease (I25.101, 2.23%), malignant neoplasm of nasopharynx (C11.901, 1.76%) and senile cataract (H25.901, 1.51%). Gender differences in the use of health care were observed under these five common conditions, although favored neither gender consistently (Table 2). Male with essential hypertension or atherosclerotic heart disease had a longer duration of hospitalization and spend more than female with the same condition (p < 0.01 for all). While female had a longer duration for other three conditions, but there was no significant difference. Moreover, the total medical expense and the daily medical cost of male were still higher than those of female under the same condition.

**Table 2:** Gender differences in variables among patients by diseases

	**Length of hospitalization, day**			**Total medical expense, yuan**			**Average daily medical expense, yuan**		
	**Male**	**Female**		**Male**	**Female**		**Male**	**Female**	
C11.901	16.77	17.05	-	11573.42	10734.77	+	907.83	843.64	+*
H25.01	4.59	4.92	-	7073.22	7063.32	+	1677.24	1664.89	+
I10 05	11.25	9.95	+**	6496.00	5234.76	+**	603.75	559.63	+**
I25.101	11.21	10.07	+**	11805.62	8159.18	+**	1073.31	825.81	+**
Z51.201	7.64	7.65	-	8327.19	7883.76	+	1357.53	1302.18	+

+:Male- Female > 0; -:Male- Female < 0.

*:*p* < 0.05; **:*p* < 0.01; *p* value refers to t-test to compare means between the two genders.

In addition, of all the subjects, 69,993 patients, accounting for 44.61%, were self-paid. Nearly half of the hospitalized women were in this setting. Of all the self-paid patients, men were superior to women in all variables investigated (Table 3). For men, the average length of hospitalization was 11.98 days, significantly longer than that observed in women (p < 0.01). The total medical expenses in men were nearly 40% more than that in women.

**Table 3:** Gender differences in variables among self-paid patients

	**Male (n = 34841)**	**Female (n = 35152)**	**Gender difference (%)**	**Sig.**
Length of hospitalization, day	11.98	9.99	19.96	0.00
Total medical expense, yuan	8823.36	6829.26	29.20	0.00
Average daily medical cost, yuan	798.08	724.73	10.12	0.00

## Discussion

This study examined the use of health-care services by gender among a city case series of patients. The results partly reflect the current situation in China that there are clear differences in the use of health care between women and men, shown by selected indicators. The proportion of male inpatients was larger than female over the seven years and across all age groups, except for the one aged from 15 to 35 years. The 20s or 30s is a prime child-bearing age. The greater use of health care in female only occurs in the childbearing years and is largely attributed to gynecological and obstetrical conditions. Regarding the duration of hospitalization and medical expense, gender differences with statistical significance were observed. Men had a longer duration of hospitalization and spent more on hospitalization compared to women. Even under the same disease condition, most differences favoring men were significant (p < 0.05) while differences favoring women were not significant (p > 0.05). In addition, gender differences in favor of male tended to be more prevalent among self-paid patients.

There are biological differences between male and female that lead to different health outcomes. It has been long-established that women live longer, but they tend to be “sicker” than men [5,14]. However this explanation is inconsistent with the disadvantage of female in health care use found in this study. In this case, the following four explanations could be better. Firstly, in this study, the median age of men (48) was 4 years older than women (44) and the proportion of patients older than 65 years was much higher in men (24.36%) than in women (16.90%). And the use of inpatient care is usually increased with the age of patient, except for a few age groups based on the age-specific health care utilization pattern [11]. Secondly, the proportion of the self-paid in female (47.05%) was higher than that in male (42.40%). According to previous researches [12,13], the self-paid made less use of health care than those with health insurances. Thirdly, most male patients were government employees who enjoyed better social welfare and health security and therefore preferred to make good use of health care. Furthermore, social factors could also be a contributor. Social structures mean that one’s gender has important health implications in terms of life position and power, access to resources and services, engagement in risk behaviors, and environmental exposures. The distinct roles and behaviors of men and women in a given culture, dictated by that culture’s gender norms and values, give rise to gender differences [15]. In China, the allocation of the resources in most families focuses on efficiency, which does not mean achieving maximum health outcomes with minimum cost but giving preferential treatment to those who can produce more economic outcome [16]. Given that women generally have less power and are poorer than men in most Chinese families, women usually postpone the maintenance of their own health after the other male household members’, especially their husbands’ and father-in-laws’. Gender norms and values, and resulting behaviors, are therefore negatively affecting the use of health care.

In addition, the preference for boys in most Chinese families also has an effect on children’s health care, which aggravates the gender differences in health. There was a high sex ratio (113.59 in 2005) at birth in Zhuhai reported by the local government [17]. Also, this study revealed the proportion of boys was higher than girls in the group aged <1. Moreover, the baby boys at this age group had a longer stay and spent more on hospitalization. In other developing countries, this phenomenon is also far from rare. A Demographic and Health Surveys program in over 40 developing countries revealed that girls were less likely to be taken to health providers than boys [18]. A new hospital in Punjab that built equal-sized wards for boys and girls found that the girls’ ward remained relatively empty, while the boys’ ward was full-filled [19].

At this point, the findings from this study have shown gender differences in the use of health care are common in China. It suggests a disadvantage of women in health care due to demographic factors as well as gender inequities. Zhuhai, as a Special Economic Zone and the “frontier” of Chinese Reform and Opening, has many modern advanced ideas, where, however, there are still gender inequities in favor of male in health care. China should increase attention to gender and equity in health. Energetic efforts should be made to promote societal change with a view to eliminate the gender barrier to good health. Gender mainstreaming policies and programmes should therefore seek to meet both men’s and women’s needs at different steps on the pathway to effective treatment. Status improvement and empowerment will improve women’s health [20].

This is the first study which applied hospitalization data to analyze gender differences in the use of health care in China. However this cross-section analysis only compared the hospitalization services. The use of health care by women might not be a constant finding but depends in part on the type of service [2]. Previous studies have reported that women tend to use preventive and diagnostic services more frequently, whereas men make greater use of emergency services [21]. In addition, the hospital delivery care is different from other inpatient cares. However, our data was unable to identify the physiological delivery or the pathological delivery (usually with complications or other diseases). We could not exclude this part while making comparison. Further studies are still needed to replicate the results found in this study. Reporting results of other on-going studies on the same topic would provide evidence for the generalizability of current findings in different settings, for other health problems, and over time. The present study does, however, serve to illustrate the association between gender and health services utilization.

## Conclusions

Gender differences in the use of health care do occur in China. Despite of demographic factors, the differences between female and male can be in part explained by social power relations and therefore inherently inequitable. China should increase attention to gender and equity in health. Future research should assess the impact of other social determinants known to cause variation in health service use that interact with gender, as well as inequities in the health care for both men and women within countries.

## Competing interests

The authors declare that they have no competing interests.

## Authors’ contributions

YS conducted the analysis and wrote the first draft of the paper. YB designed the study and reviewed all drafts of the paper. Both authors read and approved the final manuscript.
